# Estimation of Biological Parameters of Cutaneous Ulcers Caused by Leishmaniasis in an Animal Model Using Diffuse Reflectance Spectroscopy

**DOI:** 10.3390/s19214674

**Published:** 2019-10-28

**Authors:** Deivid Botina, Ricardo Franco, Javier Murillo, July Galeano, Artur Zarzycki, Maria C. Torres-Madronero, Camilo Bermúdez, Jaime Montaño, Johnson Garzón, Franck Marzani, Sara M. Robledo

**Affiliations:** 1 Research group on Advance Materials and Energy MatyEr, Biomaterials and Electromedicine Laboratory, Instituto Tecnológico Metropolitano, Calle 54A No. 30-01, Medellín 050013, Colombia; deivid.johan.botina.monsalve@gmail.com (D.B.); arturzarzycki@itm.edu.co (A.Z.); cbermudez@outlook.es (C.B.); 2 Research group on Automatic, Electronic and Computational Science, MIRP Laboratory, Instituto Tecnológico Metropolitano, Calle 54A No. 30-01, Medellín 050013, Colombia; rfrancoce@gmail.com (R.F.); mariatorres@itm.edu.co (M.C.T.-M.); 3 Program for the Study and Control of Tropical Diseases—PECET, School of Medicine, University of Antioquia, Calle 62 No. 52-59, Medellín 050010, Colombia; javier.murillo@udea.edu.co (J.M.); jaime.montano@udea.edu.co (J.M.); sara.robledo@udea.edu.co (S.M.R.); 4 Research group on Automatic, Electronic and Computational Science, Robotics and Control System Laboratory, Instituto Tecnológico Metropolitano, Calle 54A No. 30-01, Medellín 050013, Colombia; 5 Grupo de Óptica y Espectroscopía, Centro de Ciencia Básica, Universidad Pontificia Bolivariana, Cq. 1 No. 70-01, Medellín 050031, Colombia; johnson.garzon@upb.edu.co; 6 Laboratoire ImViA, Université Bourgogne Franche-Comté, B.P. 47870, 21078 Dijon CEDEX, France; franck.marzani@u-bourgogne.fr

**Keywords:** light-tissue interaction model, model inversion, cutaneous leishmaniasis, cutaneous ulcer, golden hamster

## Abstract

Cutaneous leishmaniasis (CL) is a neglected tropical disease that requires novel tools for its understanding, diagnosis, and treatment follow-up. In the cases of other cutaneous pathologies, such as cancer or cutaneous ulcers due to diabetes, optical diffuse reflectance-based tools and methods are widely used for the investigation of those illnesses. These types of tools and methods offer the possibility to develop portable diagnosis and treatment follow-up systems. In this article, we propose the use of a three-layer diffuse reflectance model for the study of the formation of cutaneous ulcers caused by CL. The proposed model together with an inverse-modeling procedure were used in the evaluation of diffuse-reflectance spectral signatures acquired from cutaneous ulcers formed in the dorsal area of 21 golden hamsters inoculated with Leishmanisis braziliensis. As result, the quantification of the model’s variables related to the main biological parameters of skin were obtained, such as: diameter and volumetric fraction of keratinocytes, collagen; volumetric fraction of hemoglobin, and oxygen saturation. Those parameters show statistically significant differences among the different stages of the CL ulcer formation. We found that these differences are coherent with histopathological manifestations reported in the literature for the main phases of CL formation.

## 1. Introduction

Leishmaniasis is a parasitic disease that can present three clinical manifestations: cutaneous, mucocutaneous, and visceral. The disease is distributed worldwide. It is present in 98 countries located especially in tropical and subtropical regions, mainly affecting developing countries. Cutaneous leishmaniasis (CL) is one of the most frequent manifestations. The World Health Organization estimates that every year 1.5 million new cases are reported worldwide [1,2]. Although CL is a public health problem, it is a neglected tropical disease and few efforts have been made towards researching novel tools that allow its understanding, diagnosis, and treatment follow-up [3]. In the most common practice, animal models are used together with bioluminescence systems for the research of new CL drugs [4]. However, those bioluminescence systems are only laboratory-use equipment that are difficult to transfer to remote areas for use in primary-level hospitals, which is where most of the human population suffering from CL are attended. For the case of other cutaneous pathologies, such as cancer or cutaneous ulcers due to diabetes, it is possible to find research works related to the use of different non-invasive tools for the investigation of those illnesses. Among those tools, those based on optical diffuse reflectance are some of the most used [5,6,7], offering portability and feasibility for the development of low-cost diagnosis and treatment follow-up systems [8].

Optical tools for diffuse reflectance data acquisition, such as spectrometers and multi-hyper/spectral imaging systems, measure the radiance reflected by a surface in hundreds or tens of spectral bands along the electromagnetic spectrum. The shape of the spectral signature allows the user to determine the materials present in a scene. The applications of this type of images are many, ranging from precision agriculture, mining, environmental studies, defense, and health. In this last area, the shape of a spectral signature could give information about the changes of a tissue when it is affected by a pathology [9]. 

When light interacts with cutaneous tissue, energy is absorbed, scattered, reflected, and transmitted due to the optical properties of the biological parameters that compose the tissue. For human tissue, there are different mathematical approaches based on diffuse reflectance models that allow estimation of a spectral signature by changing the values of the corresponding biological parameters. Among these mathematical approaches are the Kubelka–Munk model with the Fresnel equations, the radiative transfer equation, the modified Beer–Lambert law, and the photon-diffusion model [10,11,12,13,14,15,16,17]. Using a diffuse reflectance model and a minimization algorithm, an inverse-modeling procedure could be implemented. This inverse-modeling procedure allows us to estimate values for the biological parameters, such that a simulated spectral signature is very close to an acquired one. In the literature, it is possible to find inverse models with optimization algorithms such as the Levenberg–Marquardt [18,19,20] algorithm, genetic algorithms [21,22], support vector regression (SVR) [23,24], k-nearest neighbors (KNN) [25], random forest regression [26], and the Nelder–Mead simplex algorithm [27,28]. Some of the biological components that could be quantified with an inverse-modeling procedure are: melanin content, oxygen saturation, collagen concentration, hemoglobin concentration, size of collagen fibers, and skin thickness. These inverse-modeling techniques applied to measure diffuse reflectance spectral signatures have been broadly used for the analysis of some cutaneous pathologies such as vitiligo, melasma, skin cancer, and skin ulcers caused by diabetes [5,6,7,21,22]. However, in the case of cutaneous leishmaniasis, only a few efforts have been made toward the use of mathematical models for the retrieval of oxygen and hemoglobin concentration maps [17]. Therefore, there is a need to study novel mathematical techniques and experimental methodologies that could help in a better understanding of the evolution of this disease. This study could be the initial step towards the development of diffuse reflectance-based systems for CL diagnosis and treatment follow-up.

In this article, we propose the use of a three-layer diffuse reflectance model for the study of the formation of cutaneous ulcers caused by CL in an animal model. With the acquisition of spectral signatures and simulated ones, we use the Nelder–Mead simplex algorithm in order to implement an inverse model. As a result, we obtain the quantification of mathematical variables that are related to the following biological parameters: volumetric fraction of melanin, hemoglobin, and oxygen saturation; as well as diameter and volumetric fraction of keratinocytes, collagen, fibroblasts, and macrophages. These biological parameters, which are presented in the form of absorption and scattering optical variables in the diffuse reflectance model, are the most important in the different stages of CL [29]. The quantification of these parameters allows a better understanding of the evolution of CL, as an initial study of the use of spectral systems as a tool that could assist physicians in CL diagnosis and treatment follow-up.

This article is distributed as follows: in Section 2 the materials and methods are presented, which include a description of the used animal model, the type of CL, as well as the direct and inverse-modeling procedure of the light–tissue interaction model. Section 3 and Section 4 correspond to the results and conclusions, respectively.

## 2. Materials and Methods

### 2.1. Animals and Cutaneous Leishmaniasis Development

Acquisition of diffuse reflectance spectral signatures were acquired from golden hamsters. The golden hamster is recommended for CL studies, due to the similarity of the structure of its skin with respect to human skin [30,31].

A total of 21 golden hamsters (Mesocricetus auratus V strain) were used. These were distributed as 8 females and 13 males, and inoculated in their dorsal area with Leishmaniasis brasiliensis (LB). Hamsters were originally purchased from Charles River (Wilmington, MA, USA) and then raised under specific pathogen-free (spf) conditions in the PECET bioterium. Hamsters were handled according to the institutional guidelines of the Central Animal Facility at Universidad de Antioquia (Medellin, Colombia) with controlled temperature and humidity conditions, as well as food and water ad libitum [32].

For data acquisition, a mixture of ketamine (50 mg/mL) and xylazine (20 mg/mL) in a 9:1 ratio was used for anesthesia. Each animal was administered 300 µL via intraperitoneal, with a 27-gauge needle. For this, the hamster was positioned in dorsal decubitus, tilting it cranially to favor the displacement of the viscera and prevent their puncture. The aforementioned volume was slowly injected by suctioning previously in the injection site to verify that no blood comes out. 

Once the animals were anesthetized, an area of approximately 3 cm × 3 cm of fur on the dorsal skin was removed by means of a shaving device, avoiding irritation or laceration. This protocol had the approval of the Universidad de Antioquia animal ethics committee.

When the Leishmania parasite is inoculated in the skin, either experimentally or due to the bite of a sand fly, a cutaneous ulcer is formed according to the following four phases: acute phase, silent phase, active phase, and ulcerative phase. During these phases, the biological parameters or cells belonging to the epidermis and dermis present different reactions or changes [29]. Additional to the previous phases, the formation of eschar and scab layers are part of the process of development of skin ulcers. Eschar is necrotic tissue found in a wound. Blood flow in this area is poor [33]. Scab corresponds to a layer that is formed over the skin ulcer due to blood coagulation or exudate [34]. More information about the CL phases is presented in Appendix A.

### 2.2. Experimental Set-Up for Cutaneous Leishmaniasis (CL) Spectra Acquisition

**Figure 1:**
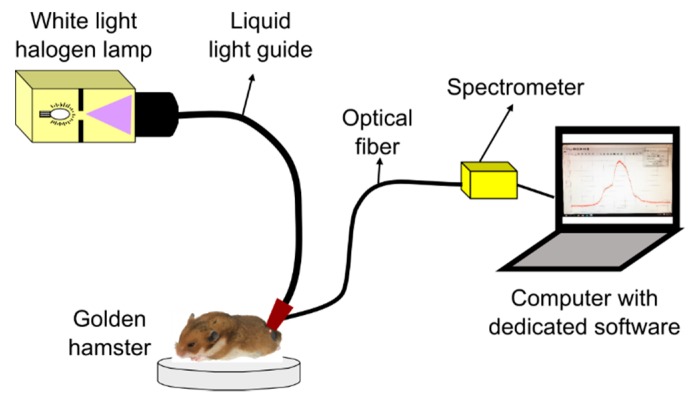
System for acquisition of diffuse reflectance spectra from healthy golden hamster skin.

Diffuse reflectance spectra from hamster dorsal skin (HDS) were acquired with a spectrometer-based system as represented in Figure 1. The system is composed of: a 150 W halogen lamp with its liquid-light guide (OSL1–High Intensity Fiber Light Source from THORLABS, Newton, NJ, USA), an optical fiber (P300-1-SR from Ocean Optics, Largo, FL, USA) connected to a spectrometer with a Charge Coupled Device (CCD) detector (HR4C3337 from Ocean Optics, Largo, FL, USA), and the dedicated software (Ocean View from Ocean Optics, Largo, FL, USA) for data acquisition. The liquid-light guide and the optical fiber were coupled to a single probe in order to avoid variability, as suggested in [35]. The probe was placed in contact with the hamster’s skin for data acquisition.

HDS was illuminated with the halogen lamp through the liquid-light guide in a collimated spot area of around 3 mm diameter. After illumination, a spectral signature was acquired through a fiber collimator lens connected to the optical fiber, which guides the reflected light to the spectrometer. The collimator lens has a numerical aperture of 0.15 and a focal distance of 18.07 mm. The optical fiber has a core diameter of 300 µm.

For each hamster, 10 diffuse reflectance spectral signatures were acquired from different areas of its dorsum. Spectra acquisition was carried out in both healthy and skin ulcer areas, in the visible-near infrared (VIS-NIR) spectral region (480 nm to 800 nm).

### 2.3. Data Acquisition and Processing Procedure

**Figure 2:**
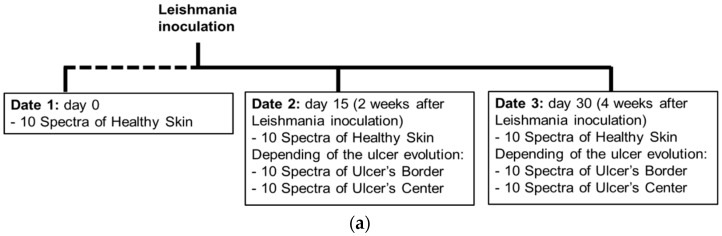

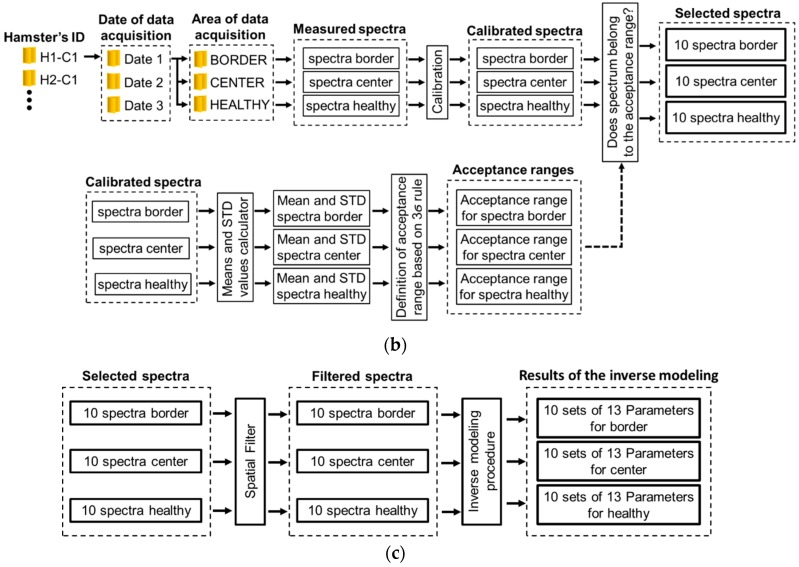
(**a**) chronology of data acquisition in golden hamster’s healthy and ulcerative skin lesion due to cutaneous leishmaniasis (CL). (**b**,**c**) procedure for processing of the acquired spectra.

As presented in the scheme of Figure 2a, data of the cutaneous ulcer formation was acquired from each hamster at the following dates: day 0, corresponding to healthy hamsters without Leishmaniasis inoculation; day 15, two weeks after inoculation; and day 30, four weeks after inoculation. At each date, 10 diffuse reflectance spectra were acquired at each one of the following regions of interest (ROI): healthy skin; border and center of the skin ulcer, depending on the CL’s evolution. The identification of healthy skin, ulcer’s border and center areas was based on the veterinary physician’s knowledge and experience.

Each ROI’s spectra acquired from each hamster at a specific date were processed according to the following procedure, which is also represented in Figure 2b,c:

(1) Spectra were calibrated by using white and black diffuse reflectance standards, which reflect more than 99% and less than 2% of light respectively (Spectralon^®^Lab Sphere, North Sutton, NH, USA). For this, diffuse reflectance spectra were acquired from the aforementioned standards in order to obtain white reference *R_w_* and black reference *R_b_* spectra. Then, the acquired skin diffuse reflectance data *R_raw_* was calibrated by means of the following Equation (1) [9]:
  (1) $$R_{m}=\frac{R_{raw}-R_{b}}{R_{w}-R_{b}}$$
  where *R_m_* is the calibrated measured spectral diffuse reflectance.
(2) Mean and standard deviation (std) values were calculated. These values were obtained from the calibrated spectra. From these values, an acceptance range of values was calculated based on the three-sigma rule (mean value +/−3std). In this way, for each hamster at each acquisition date, three acceptance range of values was calculated: for healthy skin, ulcer’s border, and ulcer’s center.
(3) Each calibrated spectrum was compared in respect to its corresponding ROI’s acceptance range, in order to verify the quality of the acquired spectrum and so detect and eliminate outlier spectra. The criteria to eliminate a spectrum was the following one: if the spectrum was outside of the three-sigma criteria range, the spectra is not considered in the experiment.
(4) Then, the accepted spectra were filtered by means of a median filter to reduce noise.
(5) Finally, each one of the filtered spectra was processed with an inverse-modeling procedure through a light–tissue interaction model and an optimization approach. After inverse modeling, 13 parameters are obtained for each one of the measured/filtered spectra. This procedure, explained in the following sub-sections, was applied to each one of the spectra acquired from the different ROI of each hamster at the different dates.

### 2.4. Direct Model: Light–Tissue Interaction Model

#### 2.4.1. Total Diffuse Reflectance

Light-tissue interaction model for diffuse reflectance of a semi-infinite turbid medium can be described by the exponential formulation presented by Equation (2) [17,36]:

(2) $$R_{t}=2\mu_{s}^{\prime }\int_{0}^{\infty }e^{-2\left(\mu_{s}^{\prime }+\mu_{a}\right)Z}dZ=\frac{\mu_{s}^{\prime }}{\mu_{s}^{\prime }+\mu_{a}},$$

where: $R_{t}$ is the total diffuse reflectance, $\mu_{s}^{\prime }$ is the reduced scattering coefficient, $\mu_{a}$ is the absorption coefficient, and Z is the thickness of the tissue. 

In order to take into account the main biological components of the three layers of hamster skin (epidermis, dermis and hypodermis), we propose a three layer model in which these biological components are represented in the form of absorption and scattering variables. So, total diffuse reflectance is given by Equation (3):

(3) $$R_{t}\left(\lambda ,\mu_{a},\mu_{s}^{\prime }\right)=R_{epi}\left(\lambda ,\mu_{a,epi},\mu_{s,epi}^{\prime }\right)+R_{epi}\left(\lambda ,\mu_{a,der},\mu_{s,der}^{\prime }\right)+R_{epi}\left(\lambda ,\mu_{a,hyp},\mu_{s,hyp}^{\prime }\right)$$

Subscripts *epi*, *der* and *hyp* correspond to the epidermis, dermis and hypodermis layers, respectively. Based on Equation (2), Equation (3) can be expressed as an equation in parts as follows (Equations (4)–(6)):

(4) $$R_{epi}=2\mu_{s,epi}^{\prime }\int_{0}^{V_{1}}e^{-2\left(\mu_{s,epi}^{\prime }+\mu_{a,epi}\right)Z}dZ,$$

(5) $$R_{der}=2\mu_{s,der}^{\prime }\int_{V_{1}}^{V_{2}}e^{-2\left(\mu_{s,der}^{\prime }+\mu_{a,der}\right)Z}dZ,$$

(6) $$R_{hyp}=2\mu_{s,hyp}^{\prime }\int_{V_{2}}^{\infty }e^{-2\left(\mu_{s,hyp}^{\prime }+\mu_{a,hyp}\right)Z}dZ,$$

where *V*_1_ is a variable related to the thickness of epidermis (6.1 µm to 6.6 µm) and *V*_2_ is related to the thickness of dermis (10 µm to 370 µm) plus epidermis in hamsters [37].

Solving Equations (4)–(6), Equations (7)–(9) are obtained:

(7) $$R_{epi}=-\frac{\mu_{s,epi}^{\prime }}{\mu_{s,epi}^{\prime }+\mu_{a,epi}}\left[e^{-2\left(\mu_{s,epi}^{\prime }+\mu_{a,epi}\right)V_{1}}-1\right],$$

(8) $$R_{der}=-\frac{\mu_{s,der}^{\prime }}{\mu_{s,der}^{\prime }+\mu_{a,der}}\left[e^{-2\left(\mu_{s,der}^{\prime }+\mu_{a,der}\right)V_{2}}-e^{-2\left(\mu_{s,der}^{\prime }+\mu_{a,der}\right)V_{1}}\right],$$

(9) $$R_{hyp}=\frac{\mu_{s,hyp}^{\prime }}{\mu_{s,hyp}^{\prime }+\mu_{a,hyp}}\left[e^{-2\left(\mu_{s,hyp}^{\prime }+\mu_{a,hyp}\right)V_{2}}\right],$$

where *R_epi_*, *R_der_* and *R_hyp_* are the contributions to the total diffuse reflectance by each one of the layers: epidermis, dermis, and hypodermis respectively; $\mu_{s,epi}^{\prime }$, $\mu_{s,der}^{\prime }$ and $\mu_{s,hyp}^{\prime }$ are the reduced scattering coefficient per layer; $\mu_{a,epi}$, $\mu_{a,der}$ and $\mu_{a,hyp}$ are the absorption coefficients per layer. Since the biological parameters responsible for light absorption and scattering in the VIS-NIR (480 nm to 800 nm) in golden hamsters’ skin are not defined, we assume an approximation with the corresponding parameters in human skin as presented in [10,16,17,38,39]. For the purposes of our work, we named those parameters in general way as variables number 3 (*V*_3_) to number 13 (*V*_13_), as they are presented in the following sub-sections.

#### 2.4.2. Absorption Coeffcients

The absorption coefficients in hamster skin are defined as follows:

(1) $\mu_{a,epi}$ represents the absorption coefficient of epidermis layer. It can be described by Equation (10):
  (10) $$\mu_{a,epi}=\mu_{a,mel}\left(\lambda \right)V_{3}+\mu_{a,back}\left(\lambda \right)\left(1-V_{3}\right),$$
  where $\mu_{a,mel}\left(\lambda \right)$ corresponds to the absorption spectrum of melanin, *V*_3_ is a variable related to the volume fraction of melanosomes, and $\mu_{a,back}$ to the background absorption, which can be described by Equation (11):
  (11) $$\mu_{a,back}=7.84\times 10^{8}\lambda^{-3.255}.$$
(2) $\mu_{a,der}$ represents the absorption component of dermis. It can be described by Equation (12):
  (12) $$\mu_{a,der}=\mu_{a,blood}\left(\lambda \right)V_{4}+\mu_{a,back}\left(\lambda \right)\left(1-V_{4}\right),$$
  where *V*_4_ is the variable related to volume fraction of blood, and $\mu_{a,blood}\left(\lambda \right)$ is the blood absorption due to oxyhemoglobin and deoxy-hemoglobin (Equation (13)):
  (13) $$\mu_{a,blood}\left(\lambda \right)=\mu_{a,oxy}\left(\lambda \right)+\mu_{a,deoxy}\left(\lambda \right)$$
  The absorption coefficient of oxyhemoglobin $\mu_{a,oxy}\left(\lambda \right)$ and deoxy-hemoglobin $\mu_{a,deoxy}\left(\lambda \right)$ are given by Equations (14) and (15), respectively:
  (14) $$\mu_{a,oxy}\left(\lambda \right)=\epsilon_{oxy}\left(\lambda \right)C_{heme}V_{5}/66500$$
  (15) $$\mu_{a,deoxy}\left(\lambda \right)=\epsilon_{deoxy}\left(\lambda \right)C_{heme}\left(1-V_{5}\right)/66500$$
  where $\epsilon_{oxy}\left(\lambda \right)$ and $\epsilon_{deoxy}\left(\lambda \right)$ are the molar extinction coefficients of oxyhemoglobin and deoxy-hemoglobin respectively [in $cm^{-1}/\left(mol/liter\right)$], with molecular weight of 66,500gram/mol. These molar extinction coefficients are found in the literature [40,41]. $C_{heme}$ is the concentration of hemoglobin in blood (150 gram/liter) [17,40]. *V*_5_ is the variable related to oxygen saturation.
(3) $\mu_{a,hyp}$, is the absorption component of hypodermis, according to Van Veen et al. [42].

Table 1 presents the absorption values considered for human skin and their related variable considered for the proposed model in hamster skin (represented in the hamster skin model as variables number 3 to 5 *V*_3_ to *V*_5_).

#### 2.4.3. Scattering Coefficients

In the case of the reduced scattering coefficients ($\mu_{s,epi}^{\prime }$, $\mu_{s,der}^{\prime }$ and $\mu_{s,hyp}^{\prime }$ at epidermis, dermis and hypodermis respectively), their values can be defined by using the Mie theory [43]. These values are estimated as a function of the refractive index n, diameter D and volume fraction VF of the scattering particle. In cutaneous tissue, these scattering particles correspond to the main cells present in each one of the tissue’s layers. Table 2 presents the diameters of the main cells that are present in each layer of human skin, represented in the hamster-skin model as variables number 6 to 9 (*V*_6_ to *V*_9_). Also, for each cell a VF is considered, which is in the range of values between 0 and 1. For hamster skin, these parameters are represented in this article as variables number 10 to 13 (*V*_10_ to *V*_13_).

### 2.5. Inverse-Modeling Procedure

The implemented inverse-modeling procedure is based on an optimization approach and the three-layer diffuse reflectance model presented in Section 2.4. The objective of the inverse-modeling procedure is to obtain a measured spectrum the corresponding parameters described in Section 2.4: absorption: variables *V*_3_ to *V*_5_, scattering: *V*_6_ to *V*_9_, and thickness of epidermis a dermis: *V*_1_ and *V*_2_, respectively.

The optimization approach used in this work corresponded to the Nelder–Mead simplex algorithm, as described by Lagarias et al. [47]. This numerical method finds the minimum of the error function between the simulated and the acquired spectra. For this purpose, the Nelder–Mead simplex algorithm examines the vertices of a simplex. This simplex is composed of n +1 points for n-dimensional vectors x with each variable. The simplex shape flip-flops towards its goal, growing, shrinking, and changing its shape according to a set of rules, until converging to the optimum solution [47].

The steps for the inverse-modeling procedure are the following ones:

(1) The variables (*V*_3_ to *V*_13_) are initialized in the middle value of the range values described in Section 2.4.2 and Section 2.4.3, Table 1 and Table 2. Variables *V*_1_ and *V*_2_ are initialized in the middle value of the range value of hamster’s epidermis and dermis thickness respectively (see Section 2.4.1).
(2) From these initial values, a simulated diffuse Reflectance (*R_s_*) is obtained by means of Equation (3).
(3) A measured diffuse reflectance signature (*R_m_*) is taken from a database with acquired/filtered spectra of golden HDS (see Section 2.3).
  Mean square error (*MSE*) of *R_m_* and *R_s_* is calculated in the Nelder–Mead simplex algorithm. *MSE* is calculated according to Equation (16) [48]:
  (16) $$MSE=\frac{1}{n}\sum_{\lambda =480}^{800}{\left(R_{m}-R_{s}\right)}^{2},$$
  where: *n* corresponds to the number of points in one diffuse reflectance spectrum. In our case, n corresponds to 321 points (800–480); *R_m_* is the vector with the measured diffuse reflectance, and *R_s_* is the simulated one.
(4) Once the *MSE* is evaluated, the procedure evaluates if this value converges to a minimum value less than 1. If so, the inverse-modeling procedure stops and the final result for the evaluated *R_m_* corresponds to the current values of the light-tissue model’s variables. Otherwise, the procedure continues to step number 6.
(5) In this step, the Nelder–Mead simplex optimization approach calculates new values for the light-tissue model’s variables. Once with these new values, the procedure returns back to step number 2.

### 2.6. Representation of the Acquired Spectra and the Inverse-Modeling Results

The results obtained from the inverse-modeling procedure were organized in the following way:

(1) As explained before, for each hamster 10 spectra were obtained from each ROI at a specific date. This means that 10 values were obtained for each one of the 13 light-tissue model’s parameters at each analyzed ROI at a specific date. From these 10 values, the median value was calculated. This means that, for each hamster at each date, 13 median values were obtained for each one of the following three ROI: healthy area, ulcer’s border and ulcer’s center.
(2) Also, from the 10 spectra acquired from the border and center of the skin ulcer at a specific date, the corresponding mean spectra were calculated.
(3) Then, from all the measured/filtered spectra at the healthy skin areas from all the hamsters, a mean spectrum was calculated. This mean spectrum is considered as a reference pattern.
(4) For each of the mean ulcers’ border and ulcers’ center spectra, a correlation coefficient was calculated in respect to the reference pattern. The correlation coefficient allows: 1—to characterize the changes of the spectral signature according to the evolution of a skin ulcer; and 2—to present the changes in the retrieved light-tissue model’s parameters, and identify characteristic groups based on an Anova analysis. The result of this procedure is presented in the following section.

## 3. Experimental Results and Discussion

### 3.1. Behavior of the Acquired and Simulated Diffuse Reflectance Spectra of Golden Hamsters’ Healthy and Ulcerated Skin

**Figure 3:**
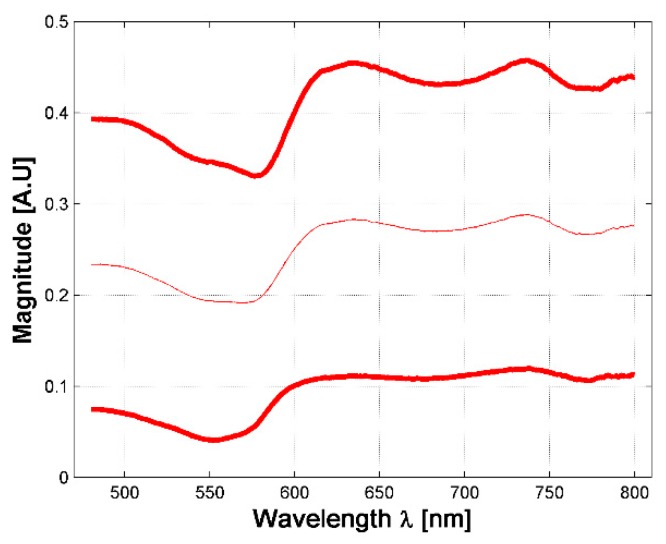
Variations in measured diffuse reflectance spectra from all hamsters. Light color line corresponds to the mean spectra. Bold lines correspond to the spectra obtained from the 3 sigma rule.

A total of 630 diffuse reflectance spectra were obtained from golden hamster healthy skin: 390 from male hamsters, and 240 from female ones. In Figure 3, the variations of the measured healthy skin spectra for all hamsters are presented. The corresponding standard deviation (STD) was calculated, so that the figure shows the variations of the mean spectrum when adding and subtracting 3 times the corresponding SDT (3 sigma rule). It is possible to observe that the acquired spectral signatures present an offset value which ranges from 0.03 up to 0.3. The same conclusion is obtained when analyzing healthy spectra of female and male hamsters individually.

**Figure 4:**
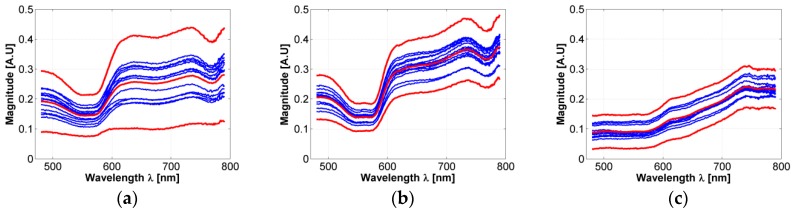

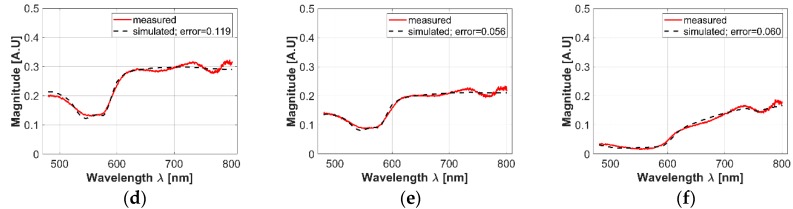
(**a**–**c**): examples of acceptance range values obtained from the acquired spectra and calculated with the 3-sigma rule. (**a**) An acceptance range for healthy skin spectra; (**b**) an acceptance range obtained from the ulcer’s border spectra; (**c**) an acceptance range obtained from ulcer’s center spectra. Light-red color line corresponds to the mean spectra. Bold red-colored lines correspond to the spectra obtained from the 3 sigma rule. Blue lines correspond to acquired spectra inside the acceptance range. (**d**–**f**): examples of simulated values after the fitting process in: (**d**) healthy skin, (**e**) ulcer’s border, and (**f**) ulcer’s center spectra.

In respect to the acceptance range value obtained from the three sigma rule (as explained in Section 2.3), Figure 4a presents an example of an acceptance range value in which a healthy-skin spectrum must belong in order to be chosen for the experiment and not to be considered as an outlier data. Also, the 3-Sigma rule was applied to the spectral signatures acquired in the borders and centers of the hamsters’ skin ulcers. Figure 4b,c present an example of the obtained acceptance ranges.

As presented in Figure 4a–c, it is possible to observe that in the case of most of the ulcer’s border area, small changes are observable in the regions between 600 to 780 nm: spectral signature magnitude is partially attenuated. These changes could be caused by the variations of collagen concentration in skin ulcers, which is a characteristic behavior during formation of dermal granulomas [29]. In the case of ulcers’ centers, the spectral signatures present a pattern with attenuated reflectance magnitude along all the spectra. Compared to the spectral behavior present in the literature for healthy human skin diffuse reflectance spectrum, the variations in the ulcer’s center spectral signatures could be caused mainly by changes in oxygen concentration.

In the case of the simulated diffuse reflectance spectra obtained from the inverse-modeling procedure, Figure 4d–f present examples of simulated values after the fitting process in healthy skin, the ulcer’s border, and the ulcer’s center spectra. In all cases, it is possible to observe that the obtained MSE values are less than 1.

### 3.2. Skin Ulcer Evolution Based on Its Final Size

Following the common practice used by physicians for the clinical evaluation of skin ulcers, data was classified according to the final size of the skin ulcer. For this study, three types of skin ulcers were identified: I—ulcers with final area of more than 95 mm^2^, II—ulcers with final area of around 50 mm^2^, and III—ulcers with a final area of around 15 mm^2^. The process of formation of a CL skin ulcer was the following: from healthy skin at the first date, to the presence of a nodule in the second (and even at the third date), until to the presence of the skin ulcer. Table A1 in Appendix B presents the identified types of skin ulcer’s formation and the corresponding hamsters.

**Figure 5:**
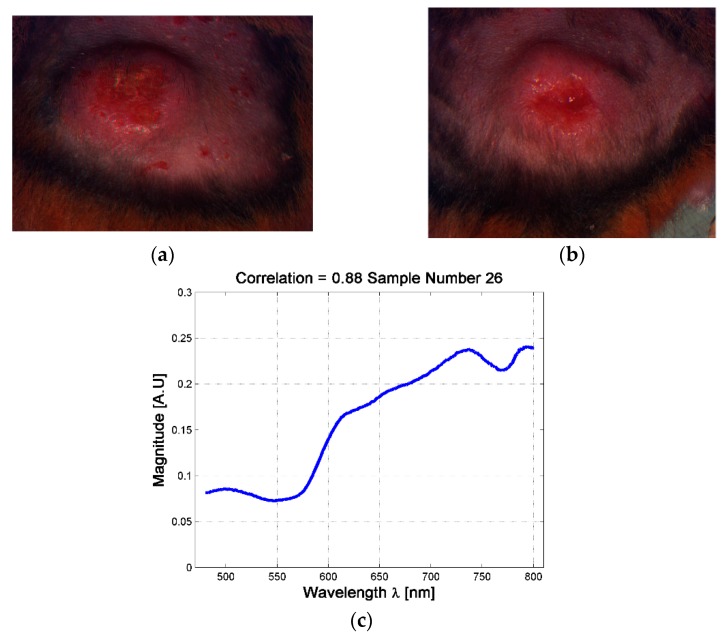
Example of skin ulcers with different sizes and similar spectral signatures at the center of the skin ulcer. (**a**) skin ulcer of Hamster 763-MAD-Date 3; (**b**) Skin ulcer of Hamster 762-MAI—Date 3; (**c**) example of the mean spectra at the center of the skin ulcer.

Although the analysis based on skin ulcer’s size is the common practice used among physicians for the analysis of skin ulcers’ evolutions [31,49], from a spectral point of view it was possible to find situations in which two skin ulcers with different size have the same spectral signature. For example, it is possible to observe the case of the skin ulcers in hamsters 763-MAD and 762-MAI on date 3 (four weeks after inoculation, see Figure 5). The ulcers have size of 40.85 mm^2^ and 71.7 mm^2^ respectively. This could indicate that although both hamsters presented skin ulcers with different external size, internally they are reacting in similar way and developing the illness in an equivalent rate. Therefore, the changes on the spectral signature could be useful as an indicator of the current stage or phase of CL skin ulcer, regardless of its size. This fact is presented in the following sub-section.

### 3.3. Changes in the Diffuse Reflectance Spectra of Golden Hamsters’ Skin during the Formation of Skin Ulcer

**Figure 6:**
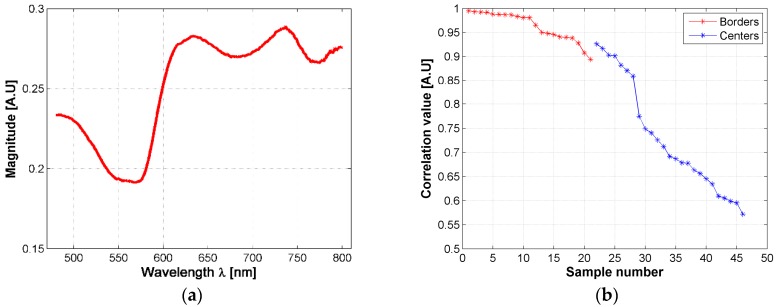
(**a**) mean diffuse reflectance of golden hamster’s healthy skin (reference pattern); (**b**) correlation coefficients obtained from the mean spectra of skin ulcer’s borders and centers.

The changes in the shape of a skin ulcer’s diffuse reflectance spectra could be related with the reactions that skin’s biological parameters present due to the presence of the Leishmania parasite. Based on the previous fact, and in order to have a better understanding of CL ulcer evolution, the skin ulcers’ mean spectra were sorted according to its similarity to healthy skin mean spectrum. This similarity was measured with a correlation coefficient as explained in Section 2.6. Both, border and center of skin ulcers’ spectra were analyzed. Results are presented in Figure 6 and the supplementary material.

Based on the correlation values, hamsters’ spectra were grouped according to their correlation value, each 0.05. So that, the spectra obtained from the ulcer’s border were divided in three groups: a first group corresponding to those spectra with a correlation value in the range (0.95, 1], a second group corresponding to those spectra with a correlation value in the range (0.90, 0.95], and a third one corresponding to [0.85, 0.90]. In the case of the spectra obtained from the ulcer’s centers, seven groups were obtained according to the following correlation ranges: (0.90, 0.95], (0.85, 0.90], (0.75, 0.80], (0.70, 0.75], (0.65, 0.70], (0.60, 0.65], (0.55, 0.60]. Then, the results of the inverse-modeling procedure were grouped in the same way. These results are presented in the following section.

### 3.4. Results from the Inverse-Modeling Procedure

**Figure 7:**
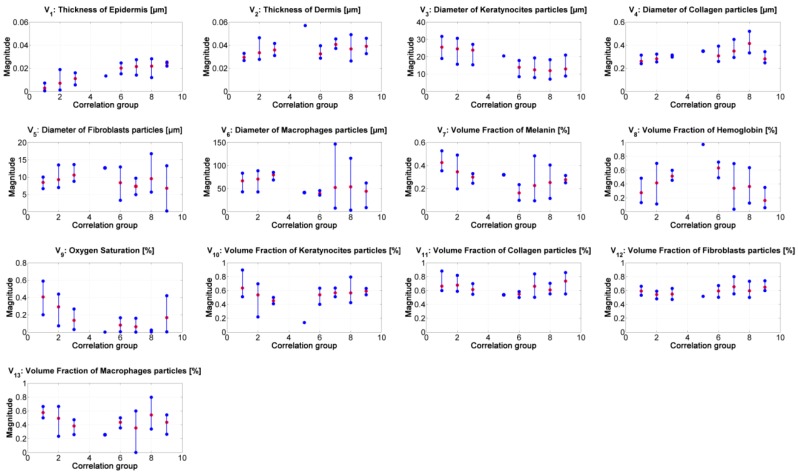
Results of the inverse modeling procedure. The results correspond to the median value obtained from the inverse model’s results obtained from the spectra belonging to the corresponding correlation-coefficient group. Red dots: median value of the corresponding samples in the group. Blue points: minimum and maximum values of the group’s results.

Figure 7 presents the median value of the absorption and scattering model’s variables obtained from the inverse-modeling applied to the spectra belonging to the specified correlation groups (as described previously). The results were sorted according to the range values obtained from the correlation.

From the results of the inverse-modeling procedure, an analysis of variance (Anova) test was carried out. With this test, it is possible to calculate *p*-values in order to identify statistical differences between the analyzed groups of absorption and scattering variables. According to the literature, there is a statistically significant difference between groups when the corresponding p-value is less than 0.05 [50]. The most relevant results of this Anova test are presented in Table 3, Table 4, Table 5, Table 6, Table 7 and Table 8. Significant values are highlighted in bolded letters. *p*-values with groups 4 and 5 are not calculated because these two groups have less than 2 samples.

**Figure 8:**
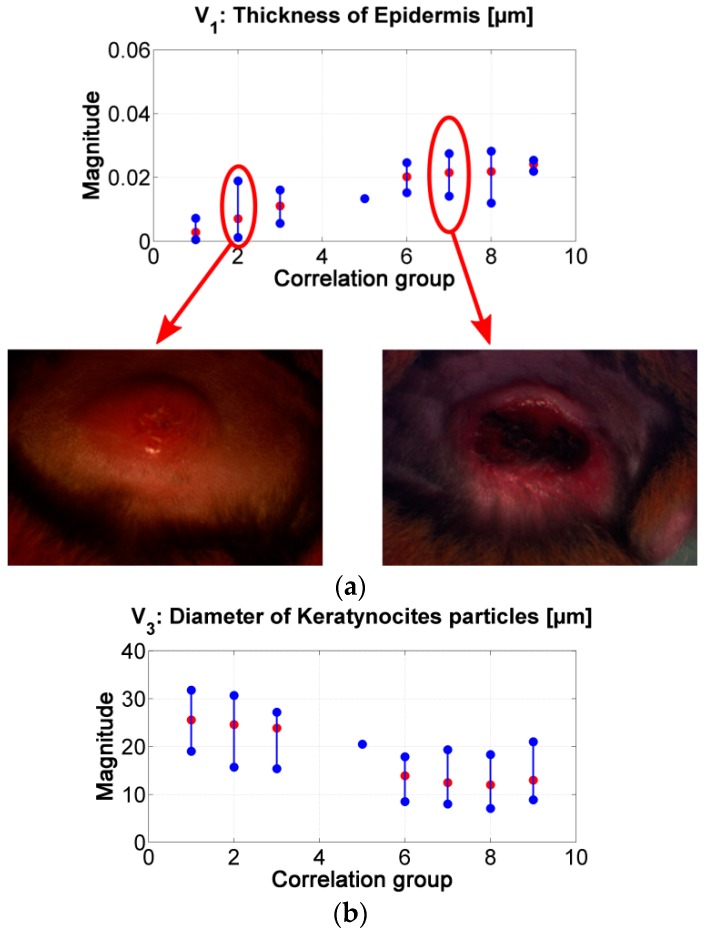
(**a**) results of the inverse-modeling procedure for variable *V*_1_: thickness of epidermis. (**b**) results of the inverse-modeling procedure for variable *V*_3_: diameter of keratynocite particles.

**Figure 9:**
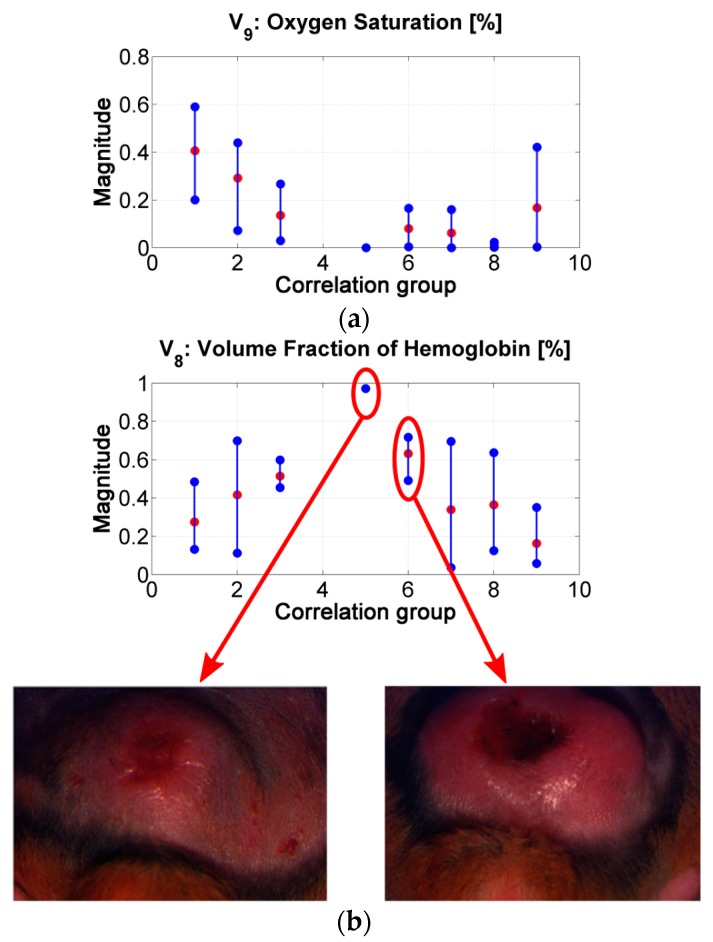
(**a**) results of the inverse-modeling procedure for variable *V*_9_: oxygen saturation. (**b**) results of the inverse-modeling procedure for variable *V*_8_: volume fraction of hemoglobin.

Similar to the results obtained from the correlation values in Figure 6b, it is possible to infer the tendency in the changes of the absorption and scattering variables during the evolution of leismaniasis skin ulcer, passing from the border to the center of skin ulcer (from the earlier stages of CL to the necrosis of the tissue). Figure 8 and Figure 9 present the relevant results.

As presented in Figure 8, the “epidermis thickness” variable (Figure 8a) is related with the thickness of the first layer in skin. Higher values can be associated to the presence of a thicker scab (see, for example, center of ulcer in range #2 vs. in range #7). The variable “keratynocites in epidermis” (Figure 8b) can follow the same analysis; lower values can be related with the presence of a more necrotic scab and so that to death cells. This behavior agrees with the transition from active phase to ulcerative one during the formation of a cutaneous ulcer caused by CL: the dermis passes from a proliferation of keratinocyte to keratinocyte cell death [29,51]. In respect to *p*-values, the results obtained for the variables “epidermis thickness” and “keratynocites in epidermis” in correlation groups 1 to 4 are statistically significantly different from those obtained in correlation groups 6 to 9.

The “oxygen saturation in epidermis” variable can follow the same analysis. Lower values can be related with the presence of a more necrotic scab and so that to death cells (see for example center of ulcer in range #2 vs. in range #7). *p*-values show that there is a statistically significant difference among the variable’s values obtained for groups 1 to 4 and those obtained for groups 6 to 8.

The “hemoglobin in epidermis” variable increases from group 1 to 5, presenting its maximum value in group number 5. Then, the value decreases along the rest of the groups. This behavior could be related to the change from scab to “necrotic” tissue (change from scab to an eschar). Example: center of ulcer in range #5 vs. in range #6. *p*-values in this variable shows statistically significant difference mainly in the results between the spectra belonging to correlation groups 1 to 3 and 6. This could confirm the transition from scab to eschar type-ulcers.

In the case of the other variables, the results corresponding to the groups 6 to 9 present high variability. This could be caused by the fact that the model is not adapted for necrotic tissue. From the point of view of light penetration in tissue, the thickness and the composition of eschars may not allow light to penetrate deeper than the first tissue’s layer. However, small changes are observable among the results corresponding to groups 1 to 5; for example, melanin variable, dermis thickness, and collagen variables. The value of these two last variables increase from group 1 to 5. The behavior of these variables could be related to inflammation and to the formation of granulomas respectively. For collagen variable, p-values show statistically significant differences between the results belonging to group 1 and groups 2 to 9.

Our results show similar behavior in case of oxygen and epidermis thickness in respect to skin ulcers caused by diabetes reported in [5]. However, those authors do not focus on the analysis of immunological cells such as fibroblasts, macrophages, as well as collagen and keratinocytes; which in the case of leishmanisis are relevant and could be differentiators in respect to other diseases.

Nevertheless, histopathological analysis of the other type of cutaneous illnesses as well as a detailed analysis of their evolution, would be necessary to address in order to determine the biological parameters that differentiate skin ulcers caused by leishmaniasis from those caused by other etiologies.

## 4. Conclusions

In this article we present the characterization of cutaneous leishmaniasis ulcer formation by means of diffuse reflectance spectra of golden hamster skin. These spectra can be modeled by means of a three-layer exponential light-interaction model, where the absorption and scattering variables represent the main biological parameters present in golden hamster skin. Absorption variables are related to: volumetric fraction of melanin, hemoglobin, and oxygen saturation. Scattering variables are related to: diameter and volumetric fraction of keratinocytes, collagen, fibroblasts, and macrophages. Also, the epidermis and dermis thicknesses are considered.

Diffuse reflectance spectra were acquired from 21 golden hamsters inoculated with L. brasiliensis. Spectra were acquired from healthy skin, as well as from the border and center of skin ulcers during different stages of CL formation. These spectra were analyzed by means of an inverse-modeling procedure, based on the Nelder–Mead optimization approach. In this inverse-modeling procedure, the model’s variables were initialized to the middle value of the reference range values of the absorption and scattering biological parameters reported in the literature for human skin. As result, the corresponding absorption and scattering variables of each measured spectrum were obtained.

We observed that the changes of the model’s variables corresponding to healthy, infected, and damaged skin are congruent with the biological phenomena present during the active and ulcerative phases of cutaneous leishmaniasis. The results obtained show a chronology in the change of the absorption and scattering variables during the evolution of Leismaniasis skin ulcer formation, passing from the border to the center of the skin ulcers. For variable 1, related to epidermis thickness, it is possible to observe an increment in its value when its corresponding diffuse reflectance spectra has a lower correlation value in respect to healthy skin spectrum (i.e., passing from the ulcer’s border to the ulcer’s center spectra). The variable related to diameter of collagen presents the same behavior. On the other hand, for variables oxygen saturation and diameter of keratinocytes, the relation between their values and the corresponding spectra correlation is direct: while the diffuse reflectance spectra are less correlated to healthy skin spectrum, the values of the variables are lower. The values obtained for the variable hemoglobin in epidermis present a behavior which could be related to the change from scab to “necrotic” tissue (change from scab to an eschar) in the center of skin ulcers. 

The behaviors described could all be related to the histopathological manifestation reported in the literature for the active and ulcerative phases of CL formation. In these phases, changes in keratinocytes, the presence of granulomas, and necrotic areas are relevant parameters. This shows the potential that diffuse reflectance systems have as a tool for CL characterization, as well as for the objective CL treatment follow-up. Future work will seek to characterize each stage during cutaneous leishmanisis ulcer healing.

## Figures and Tables

**Table 1:** Absorption parameters in human skin and related variable in the hamster skin’s direct model.

Biological Parameter	Skin Layer	Range Value in Human Skin	Reference	Variable in Hamster Skin’s Direct Model
Volume Fraction (V.F.) Melanosomes [%]	Dermis	0.01–0.43	[16]	*V*_3_
Volume Fraction (V.F.) Hemoglobin [%]	Dermis	0.002–0.07	[16,40]	*V*_4_
Oxygen Saturation [%]	Dermis	0–1	[16]	*V*_5_

**Table 2:** Diameter of cells in human skin and related variable in the Hamster Skin’s Direct Model.

Cell	Skin Layer	Diameter in Human Skin [µm]	Reference	Variable in Hamster Skin’s Direct Model
Keratinocytes	Epidermis	15–50	[44]	*V*_6_
Collagen	Dermis	0.03–0.3	[45]	*V*_7_
Fibroblasts	Dermis	10–15	[46]	*V*_8_
Macrophages	Dermis	20–80	[46]	*V*_9_

**Table 3:** *p*-values between the median results of variable *V*_1_ “epidermis thickness” obtained from the spectra of the corresponding correlation groups.

		Correlation Groups						
Correlation Groups		1	2	3	6	7	8	9
	1	1.000	0.341	0.081	**0.000**	**0.000**	**0.003**	**0.000**
	2	0.341	1.000	0.478	**0.006**	**0.001**	**0.015**	**0.001**
	3	0.081	0.478	1.000	**0.002**	**0.001**	**0.009**	**0.000**
	6	**0.000**	**0.006**	**0.002**	1.000	0.667	0.944	0.079
	7	**0.000**	**0.001**	**0.001**	0.667	1.000	0.681	0.309
	8	**0.003**	**0.015**	**0.009**	0.944	0.681	1.000	0.174
	9	**0.000**	**0.001**	**0.000**	0.079	0.309	0.174	1.000

**Table 4:** *p*-values between the median results of variable *V*_6_ “keratinocyte diameter” obtained from the spectra of the corresponding correlation groups.

		Correlation Groups						
		1	2	3	6	7	8	9
Correlation Groups	1	1.000	0.456	**0.027**	**0.001**	**0.001**	**0.002**	**0.003**
	2	0.456	1.000	0.085	**0.002**	**0.001**	**0.003**	**0.005**
	3	**0.027**	0.085	1.000	**0.002**	**0.002**	**0.003**	**0.006**
	6	**0.001**	**0.002**	**0.002**	1.000	0.464	0.708	0.563
	7	**0.001**	**0.001**	**0.002**	0.464	1.000	0.735	0.951
	8	**0.002**	**0.003**	**0.003**	0.708	0.735	1.000	0.809
	9	**0.003**	**0.005**	**0.006**	0.563	0.951	0.809	1.000

**Table 5:** *p*-values between the median results of variable *V*_9_ “oxygen saturation” obtained from the spectra of the corresponding correlation groups.

		Correlation Groups						
		1	2	3	6	7	8	9
Correlation Groups	1	1.000	0.446	**0.013**	**0.002**	**0.000**	**0.000**	**0.065**
	2	0.446	1.000	**0.005**	**0.000**	**0.000**	**0.000**	**0.043**
	3	**0.013**	**0.005**	1.000	0.145	0.051	**0.018**	0.628
	6	**0.002**	**0.000**	0.145	1.000	0.560	0.078	0.639
	7	**0.000**	**0.000**	0.051	0.560	1.000	0.255	0.467
	8	**0.000**	**0.000**	**0.018**	0.078	0.255	1.000	0.257
	9	0.065	**0.043**	0.628	0.639	0.467	0.257	1.000

**Table 6:** *p*-values between the mean results of variable *V*_4_ “collagen diameter” obtained from the spectra of the corresponding correlation groups.

		Correlation Groups						
		1	2	3	6	7	8	9
Correlation Groups	1	1.000	**0.001**	**0.022**	0.094	**0.003**	**0.000**	0.125
	2	**0.001**	1.000	0.463	0.326	**0.027**	**0.001**	0.496
	3	**0.022**	0.463	1.000	0.377	0.094	**0.002**	0.565
	6	0.094	0.326	0.377	1.000	0.820	**0.015**	0.743
	7	**0.003**	**0.027**	0.094	0.820	1.000	**0.009**	0.517
	8	**0.000**	**0.001**	**0.002**	**0.015**	**0.009**	1.000	**0.007**
	9	0.125	0.496	0.565	0.743	0.517	**0.007**	1.000

**Table 7:** *p*-values between the mean results of variable *V*_8_ “hemoglobin” obtained from the spectra of the corresponding correlation groups.

		Correlation Groups						
		1	2	3	6	7	8	9
Correlation Groups	1	1.000	0.145	0.059	**0.007**	0.914	0.486	0.050
	2	0.145	1.000	0.729	**0.047**	0.538	0.719	0.070
	3	0.059	0.729	1.000	**0.007**	0.382	0.850	**0.008**
	6	**0.007**	**0.047**	**0.007**	1.000	0.152	0.149	**0.001**
	7	0.914	0.538	0.382	0.152	1.000	0.800	0.163
	8	0.486	0.719	0.850	0.149	0.800	1.000	0.061
	9	0.050	0.070	**0.008**	**0.001**	0.163	0.061	1.000

**Table 8:** *p*-values between the mean results of variable *V*_7_ “melanin” obtained from the spectra of the corresponding correlation groups.

		Correlation Groups						
		1	2	3	6	7	8	9
Correlation Groups	1	1.000	**0.003**	**0.036**	**0.000**	**0.019**	**0.044**	**0.004**
	2	**0.003**	1.000	0.699	**0.003**	0.215	0.211	0.050
	3	**0.036**	0.699	1.000	**0.002**	0.168	0.144	**0.026**
	6	**0.000**	**0.003**	**0.002**	1.000	0.233	0.406	0.082
	7	**0.019**	0.215	0.168	0.233	1.000	0.757	0.801
	8	**0.044**	0.211	0.144	0.406	0.757	1.000	0.877
	9	**0.004**	0.050	**0.026**	0.082	0.801	0.877	1.000

## Supplementary Materials

The following are available online at https://www.mdpi.com/1424-8220/19/21/4674/s1, Table S1: The results of border and center of skin ulcers’ spectral analysis.

## Appendix A

### Phases of Cutaneous Leishmaniasis Development

Once the Leishmania parasite is inoculated in the skin, either in experimental way or due to the bite of a sand fly, a cutaneous ulcer is formed according to the following four phases: acute phase, silent phase, active phase, and ulcerative phase. During these phases, the biological parameters or cells belonging to epidermis and dermis present different reactions or changes. Among those reactions, it is possible to name the following ones [29]:

1. Acute phase: no changes are present in epidermis. In the case of dermis, an inflammatory reaction is present due to the infiltration of neutrophils, dendritic cells, and monocyte cells.
2. Silent phase: no changes are present in epidermis. In the case of dermis, the highest parasite load is present as well as an inflammatory infiltrate due to monocytes/macrophages.
3. Active phase: a proliferation of keratinocytes occurs in the epidermis. For dermis, a sign of necrotic areas with apoptotic neutrophils is present; also, the infiltration of immunological cells, such us T cells and Langerhans cells, is present.
4. Ulcerative phase: keratinocyte cell death is present in the epidermis. In the dermis, dermal granulomas are present together with neutrophils, as well as an increase of infiltration of immunological cells such as B and plasma cells.

## Appendix B

**Table A1:** Types of skin ulcer’s formation according to its final size.

Type of Ulcer Development	Description of the Skin Ulcer Formation	ID Hamsters	
		Female	Male
I	Healthy-nodule-big ulcer with border and more than *95 mm^2^* area.	762-MPD	764-MPD
		763-MAI	764-MPI
		756-MPD	766-MAI
			755-MAD
			764-MAD
			758-MAI
			759-MAI
II	Healthy-nodule-middle ulcer with border and size around *50 mm^2^* area.	762-MAI	758-MPD
		763-MAD	755-MPD
		763-MPD	764-MAI
		756-MAI	766-MAD
			766-MPD
III	Healthy-nodule-small ulcer with border and size around *14 mm^2^* area.	756-MAD	755-MAI
